# Targeting KAT7 inhibits the progression of colorectal cancer

**DOI:** 10.7150/thno.106085

**Published:** 2025-01-02

**Authors:** Hao Wang, Tianwang Guan, Rong Hu, Zhongjie Huang, Zhao Liang, Xiaonan Lin, Yingqi Qiu, Peiyun Liao, Xiongbo Guo, Yushen Ke, Honghao Zhang, Caiwen Ou, Yuhua Li

**Affiliations:** 1 Department of Hematology, Zhujiang Hospital, Southern Medical University, Guangzhou, 510280, China.; 2 Guangdong Engineering Research Center of Precision Immune Cell Therapy Technology, Guangzhou, 510280, China.; 3 Cancer Center, The 10th Affiliated Hospital of Southern Medical University (Dongguan People's Hospital), Southern Medical University, Guangdong, 523059, China.; 4 Guangdong Provincial Key Laboratory of Cardiac Function and Microcirculation, Guangzhou, 510280, China.; 5 Dongguan Institute of Clinical Cancer Research, Dongguan Key Laboratory of Precision Diagnosis and Treatment for Tumors, Dongguan Engineering Research Center for Innovative Boron Drugs and Novel Radioimmune Drugs, The Tenth Affiliated Hospital, Southern Medical University (Dongguan People's Hospital), Dongguan, 523059, China.; 6 The 10th Affiliated Hospital of Southern Medical University (Dongguan People's Hospital), Southern Medical University, Guangdong, 523059, China.; 7 Department of Gastrointestinal Surgery, The Second Affiliated Hospital of Guangzhou Medical University, Guangzhou, 510280, China.

**Keywords:** colorectal cancer, target therapy, MAPK, epigenetics, KAT7

## Abstract

**Rationale:** Colorectal cancer (CRC) is a leading cause of cancer-related mortality. Epigenetic modifications play a significant role in the progression of CRC. KAT7, a histone acetyltransferase, has an unclear role in CRC.

**Methods:** In this research, we analyzed the expression of KAT7 in CRC patients and its correlation with prognosis using the GEO database, western blot, and immunohistochemistry. We assessed the impact of KAT7 on CRC cell functions through cell viability, colony formation, flow cytometry, scratch, and transwell assays. Mechanistic insights were obtained via RNA sequencing and ChIP-qPCR. Additionally, we evaluated the effects of KAT7 on CRC growth and metastasis *in vivo* using mouse subcutaneous tumor and lung metastasis models.

**Results:** In this study, we discovered an upregulated KAT7 signaling pathway in CRC and its association with poor patient survival. Knockdown of KAT7 promotes apoptosis and inhibits proliferation, migration, and invasion of CRC cells. Conversely, KAT7 overexpression enhanced these cellular processes. *In vivo* assays confirmed that knockdown of KAT7 can inhibit CRC proliferation and lung metastasis. Mechanistically, KAT7 acetylated histone H3 at lysine 14 (H3K14) to enhance MRAS transcription, which activated the MAPK/ERK pathway and promoted tumorigenesis. The enzymatic function of KAT7 as an acetyltransferase is crucial for the advancement of colorectal cancer. In KAT7 knockdown CRC cells, re-expression of KAT7, but not an acetyltransferase-deficient mutant, rescued MRAS expression, ERK phosphorylation, and CRC tumorigenesis.

**Conclusion:** We found that KAT7 is highly expressed in CRC patients, and those with high KAT7 expression have a worse prognosis. KAT7 enhances MRAS gene transcription by promoting H3K14 acetylation, thereby activating the MAPK/ERK pathway and promoting malignant phenotypes of CRC. In summary, KAT7 represents a promising target for CRC therapy.

## Introduction

CRC accounts for about 10% of all cancer cases and deaths globally 1. In recent years, there has been a concerning rise in CRC cases among individuals under the age of 45 2. Despite significant advancements in treatments such as radiotherapy, molecular targeted therapy, and chemotherapy, improvements in the 5-year survival rate for advanced CRC remain unsatisfactory. Specifically, the survival rate for individuals aged over 50 decreased by 34% between 2000 and 2014, while it increased by 13% for those under 50 years old 3. One major factor contributing to this slow progress is that 90% of CRC patients exhibit drug resistance 4,5. Consequently, recent studies have focused on exploring novel molecular pathogenesis and targeted therapeutics for CRC 6-11.

Research indicates that epigenetic processes, including modifications like histone acetylation and methylation, are pivotal in the progression of CRC 12-14. Among the various epigenetic alterations, the reversible acetylation of histones is essential for controlling gene expression 15-17. Two types of enzymes, histone acetyltransferases (HATs) and histone deacetylases (HDACs), primarily control histone acetylation 18,19.

KAT7 is a HAT enzyme involved in the modification of H3 and H4 histones 20-22. It is essential for gene expression, immune system regulation, stem cell pluripotency, and maintenance of self-renewal. To carry out its acetyltransferase activity, KAT7 forms complexes with scaffold proteins such as BRPF or JADE. These complexes are necessary because KAT7 lacks its chromatin binding regions. The KAT7-BRPF complex primarily targets histone H3 tails, specifically lysine 14 (K14) and lysine 23 (K23), for acetylation 20,21,23. Conversely, the KAT7-JADE complex primarily targets histone H4 tails for acetylation, specifically at lysine 5 (K5), lysine 8 (K8), and lysine 12 (K12) 22,24

Recent studies have implicated KAT7 in cancer progression. Yang *et al.* demonstrated the pivotal function of KAT7 in the early stages of lung adenocarcinoma by illustrating its interaction with the NLRP11 protein for Vimentin acetylation 25. The aggressive development of liver cancer precursors relies on the acetylation and cytoplasmic translocation of G protein GαS by KAT7 26. Patients with elevated KAT7 expression have a worse prognosis in head and neck squamous carcinoma. This is due to KAT7's function in increasing the acetylation of LDHA, which in turn boosts both the proliferation and metastasis of cancer cells 27. Some studies have suggested that KAT7 plays a critical role in the survival of leukemia cells 21,24,28. However, studies by Sauer *et al.* revealed that KAT7 expression is decreased in acute myeloid leukemia, and the lack of KAT7 promotes leukemic cell proliferation 29. Kueh and collaborators found that in breast adenocarcinoma and cervical carcinoma cells, KAT7 is non-essential for cell proliferation 30. Research on colorectal cancer indicated that the regulation of TUSC3 expression by the UHRF1-KAT7 complex via histone methylation and acetylation is crucial for the growth of colon cancer cells 31. Nonetheless, the precise function and mechanisms of KAT7 in CRC progression need additional exploration.

In this research, we began by investigating KAT7 expression in CRC cells and patient samples, as well as its correlation with patient prognosis. Subsequently, we assessed the role of KAT7 in CRC cell growth, apoptosis, movement, and invasiveness. Finally, mechanistic studies demonstrated that KAT7 facilitates MRAS gene transcription by enhancing H3K14 acetylation, thereby driving the activation of the MAPK/ERK signaling pathway and promoting the malignant phenotype of CRC.

## Methods

### Chemicals and reagents

The CCK8 reagent was sourced from Beyotime (Shanghai, China). Puromycin was purchased from Solarbio (Beijing, China), and Blasticidin was acquired from MedChemExpress (Shanghai, China). The transfection agent Lipofectamine 3000 was supplied by Thermo-Fisher Invitrogen (Shanghai, China). The antibodies employed in this research are compiled in **Table S1**.

### Cell lines and culture conditions

The human normal colonic epithelial cell line NCM460 and CRC cell lines (SW480, HCT116, CACO2, COLO320, RKO, HCT15, HT29, SW620) were cultured and maintained in our laboratory. All cell lines were cultured in DMEM with 10% fetal bovine serum. They were kept at 37°C in an atmosphere of 5% CO2.

### Human tissue samples

The tissue samples utilized in this research were collected from CRC patients who received treatment at Zhujiang Hospital in Guangzhou, China. Informed consent was secured from all participants prior to the start of the experiment. The study involving human samples received approval from Zhujiang Hospital's Institutional Review Board.

### qRT-PCR

Total RNA was isolated using TRIzol reagent (Invitrogen, USA) and quantified with a NanoDrop spectrophotometer (Thermo Scientific, USA). cDNA synthesis was performed with the PrimeScript RT Reagent Kit (Takara, Japan). qRT-PCR was conducted using SYBR Green Master Mix (Applied Biosystems, USA) on an ABI 7500 system. Reactions (20 µL) included 10 µL of SYBR Green, 1 µL of cDNA, 0.4 µL of each primer (10 µM), and 8.2 µL of water. Cycling conditions were 95°C for 10 min, then 40 cycles of 95°C for 15 sec and 60°C for 1 min. Expression levels were normalized to β-actin using the 2^(-ΔΔCt) method. The primer sequences used in this study are summarized in **Table S2**.

### Western blot analysis

As described 32, Cells were lysed in RIPA buffer with protease and phosphatase inhibitors. Protein concentration was measured using the BCA assay (Thermo Scientific, USA). Equal protein amounts (30 µg) were separated via SDS-PAGE and transferred to PVDF membranes. Membranes were blocked with 5% non-fat milk in TBS-T for 1 hour, then incubated overnight at 4°C with primary antibodies. After washing, membranes were treated with HRP-conjugated secondary antibodies for 1 hour. Bands were detected using an ECL substrate and imaged with a chemiluminescent system.

### KAT7 shRNA

Two distinct shRNAs targeting human KAT7, named shKAT7-1 (target sequence: CTGTCACCTGATTGGATATTT) and shKAT7-2 (target sequence: GCTCAAATACTGGAAGGGAAA), along with a scrambled control shRNA (“shC”, target sequence: TTCTCCGAACGTGTCACGT), were created and validated by Genechem (Shanghai, China). These shRNAs were subsequently subcloned into the GV493 vector from Genechem. The plasmids were individually transfected into HEK-293T cells using a mixture of lentiviral packaging plasmids provided by Genechem. In 24-well plates, CRC cells were plated at a concentration of 1×10^5^ cells per well. Polybrene was included in the complete culture medium to enhance transfection efficiency. Lentiviruses carrying shRNA or control were added to the culture medium of CRC cells. After one day, the medium was switched to a fresh complete medium. 72 hours after transfection, puromycin (2.0 µg/mL) was introduced into the medium, and the cells were grown for 10 days to obtain stable transfectants.

### KAT7 knockout

Two single guide RNAs (sgRNAs) targeting the human KAT7 gene (sgKAT7-1: GATGAACGAGTCTGCCGAAG; sgKAT7-2: AACGATACTCCGCCGGCACA) or a control vector (sgNC: GTATTACTGATATTGGTGGG) were cloned into a lenti-CRISPR-GFP vector. sgRNA was transfected into CRC cells using Lipofectamine 3000. GFP-positive cells were sorted by flow cytometry and subjected to single-cell cloning 72 hours post-transfection. Stable KAT7 knockout cells were identified using western blot analysis.

### Ectopic KAT7 overexpression

To achieve KAT7 overexpression, lentiviruses were obtained from Genechem. CRC cells were plated in 24-well plates at a concentration of 1×10⁵ cells per well in a complete medium. To improve transfection efficiency, Polybrene was incorporated into the medium before adding the lentiviruses. 72 hours post-transduction, the cells underwent selection with blasticidin (3 µg/mL) for 10 days to establish stable overexpression lines.

### Cell viability and colony formation assays

For assessing cell viability, cells subjected to different treatments were plated in 96-well plates with 3000 cells per well. Next, 10 µL of CCK-8 reagent was introduced to each well, followed by a 4-hour incubation period. The optical density of each well was recorded at 450 nm using a plate reader. In the colony formation assay, CRC cells subjected to different treatments were distributed into 6-well plates, each containing 1000 cells per well. After incubating for 14 days, the colonies were stained using a 1% crystal violet solution.

### Flow Cytometry (FACS) analysis

Flow cytometry was performed to assess apoptosis in CRC cells. Genetically modified or treated CRC cells were seeded into six-well plates at a density of 2 × 10⁵ cells per well and cultured under standard conditions. After treatment, cells were harvested, the complete medium was removed, and the cells were washed twice with PBS. Subsequently, the cells were stained with Annexin V and propidium iodide (PI) using an apoptosis detection kit (KeyGEN BioTECH). Stained cells were analyzed by flow cytometry using a Beckman Coulter system (Brea, CA) within 1 hour. Annexin V-positive cells were gated and identified as apoptotic cells.

### EdU (5-ethynyl-20-deoxyuridine) staining

Cell growth was measured utilizing the EdU staining method with the EdU Apollo-488/567 kit (RiboBio, Guangzhou, China). Following the protocol provided by the reagent kit, cells were incubated with EdU for an additional 2 hours. The cell nuclei were then stained with Hoechst 33342 fluorescent dye. Finally, the staining was observed under an inverted fluorescence microscope (Nikon, Shanghai, China). The cell proliferation ratio was calculated by selecting three random fields of view, covering a total of over 500 cells.

### Transwell assay

For the cell migration assay, trypsinized CRC cells were resuspended in a serum-free medium. The pore size of the transwell inserts was 8 µm (BD Biosciences, Shanghai, China). CRC cell suspensions with different treatments were plated in the upper chamber at a concentration of 30,000 cells per well, with the lower chamber filled with a complete medium supplemented with 10% FBS. After allowing cell migration for 16 hours, the inserts were taken out, and the non-migrated cells in the upper chamber were removed. The cells that had moved to the lower chamber were colored with 1% crystal violet and examined under a microscope for counting. For the cell invasion assay, a layer of Matrigel (Sigma, Shanghai, China) was required to be coated in the upper chamber.

### Wound healing assay

CRC cells subjected to different treatments were distributed into 6-well plates. Once the cells reached around 90% confluency, a sterile 200 µL pipette tip was employed to make a scratch in the cell monolayer, and the dislodged cells were washed away with PBS. The cells were subsequently cultured for another 24 hours in a serum-free medium. To establish baseline images, the cell plates were imaged using an inverted microscope at 0 hours after the scratch was made. Images were captured in triplicate for each condition. The final images were obtained at the 24-hour time point, which served as the end-point of the assay.

### Overexpression plasmids and cell transfection

The MRAS, KAT7, and KAT7-G485A plasmids were sourced from IGE. CRC cells were seeded in 6-well plates at a concentration of 1×10^6^ cells per well in a complete medium. Transfection of these plasmids was performed using Lipofectamine 3000.

### RNA-seq

Extraction of total RNA from HCT116-shC and HCT116-shKAT7-1 cells was performed using TRIzol. Total RNA (1 μg per sample) was used for library preparation using the Hieff NGS Ultima Dual-mode mRNA Library Prep Kit for Illumina (Yeasen Biotechnology, Shanghai). Following the manufacturer's recommendations, index codes were added to each sample. mRNA was purified using poly-T oligo-attached magnetic beads. First and second strand cDNA synthesis was performed, and overhangs were converted into blunt ends. After adenylation of 3' ends, the NEBNext Adaptor was ligated. Library fragments were purified using the AMPure XP system (Beckman Coulter). USER Enzyme (NEB) treatment was applied, followed by PCR with Phusion High-Fidelity DNA polymerase, Universal PCR primers, and Index primers. Final PCR products were purified and quality assessed on an Agilent Bioanalyzer 2100. Sequencing was conducted on an Illumina NovaSeq platform to generate 150 bp paired-end reads. Differential expressions with p values less than 0.05 and log2 fold change more than 1.0 were considered to be statistically significant. The data are available from the NIH GEO database (GSE270298).

### Xenograft assays

NOD.CB17-Prkdcscid/l2rgtm1/Bcgen (B-NDG) mice (4-6 weeks old) were acquired from Bes Test (Zhuhai, China). COLO320 cells (5×10^6^) with or without KAT7 knockdown were injected subcutaneously into the flanks of mice. The size of the subcutaneous tumors was assessed and calculated every 5 days using calipers, with the tumor volume calculated as (length × width^2^)/2. On day 25 post-injection, the mice were sacrificed, and the subcutaneous tumors were harvested for further experiments. For the CRC lung metastasis model, COLO320-shKAT7 or COLO320-shC cells (2×10^5^) were administered via the tail vein of B-NDG mice. After 30 days, the mice were euthanized, and lungs were harvested to evaluate tumor metastasis (n=7 per group). Additionally, survival times were monitored for mice receiving tail vein injections of COLO320-shC or COLO320-shKAT7 cells (n=10 per group). All animal experiments received approval from the Animal Research Ethics Committee of Zhujiang Hospital.

### Chromatin immunoprecipitation (ChIP) assay

The cells were first fixed with 1% formaldehyde and then quenched with a glycine solution. After that, the cells were washed and lysed with a lysis buffer. The lysate samples were subjected to fragmentation of genomic DNA (200-800 bp) using a Misonix Sonicator 3000 Homogenizer. After centrifugation, the supernatant was carefully removed, and ChIP Dilution Buffer was subsequently introduced, along with an anti-KAT7 or anti-H3K14ac antibody. Finally, the DNA bound to KAT7/H3K14ac was eluted using an elution buffer, and NaCl was introduced to separate the protein-DNA cross-links. Primers targeting the promoter region of the MRAS gene were designed and synthesized, and then the amount of MRAS promoter DNA in each treatment group was detected via PCR. The primer sequences are included in **Table S2**.

### Immunohistochemistry

Tissue samples were fixed in 10% formalin, embedded in paraffin, and sectioned at 4 µm. Sections were deparaffinized, rehydrated, and subjected to antigen retrieval in citrate buffer (pH 6.0). After blocking endogenous peroxidase with 3% hydrogen peroxide and non-specific sites with 5% BSA, primary antibodies were applied and incubated overnight at 4°C. Sections were then incubated with biotinylated secondary antibodies, followed by an avidin-biotin complex. Visualization was performed with a DAB substrate, and slides were counterstained with hematoxylin, dehydrated, cleared, and coverslipped. Images were captured using a light microscope.

### Statistical analysis

Data analysis was performed using GraphPad Prism 9 software, with results expressed as mean ± standard deviation (SD). For statistical evaluation, t-tests were used for comparing two groups, while ANOVA followed by Dunnett's test was applied for multiple group comparisons. Survival data were assessed using the Kaplan-Meier method, with comparisons made via the log-rank test. Statistical significance levels were denoted as follows: *P < 0.05, **P < 0.01, ***P < 0.001, ****P < 0.0001, or labeled as non-significant (ns) if no significance was observed.

## Results

### KAT7 expression is significantly up-regulated in CRC

**Figure 1:**
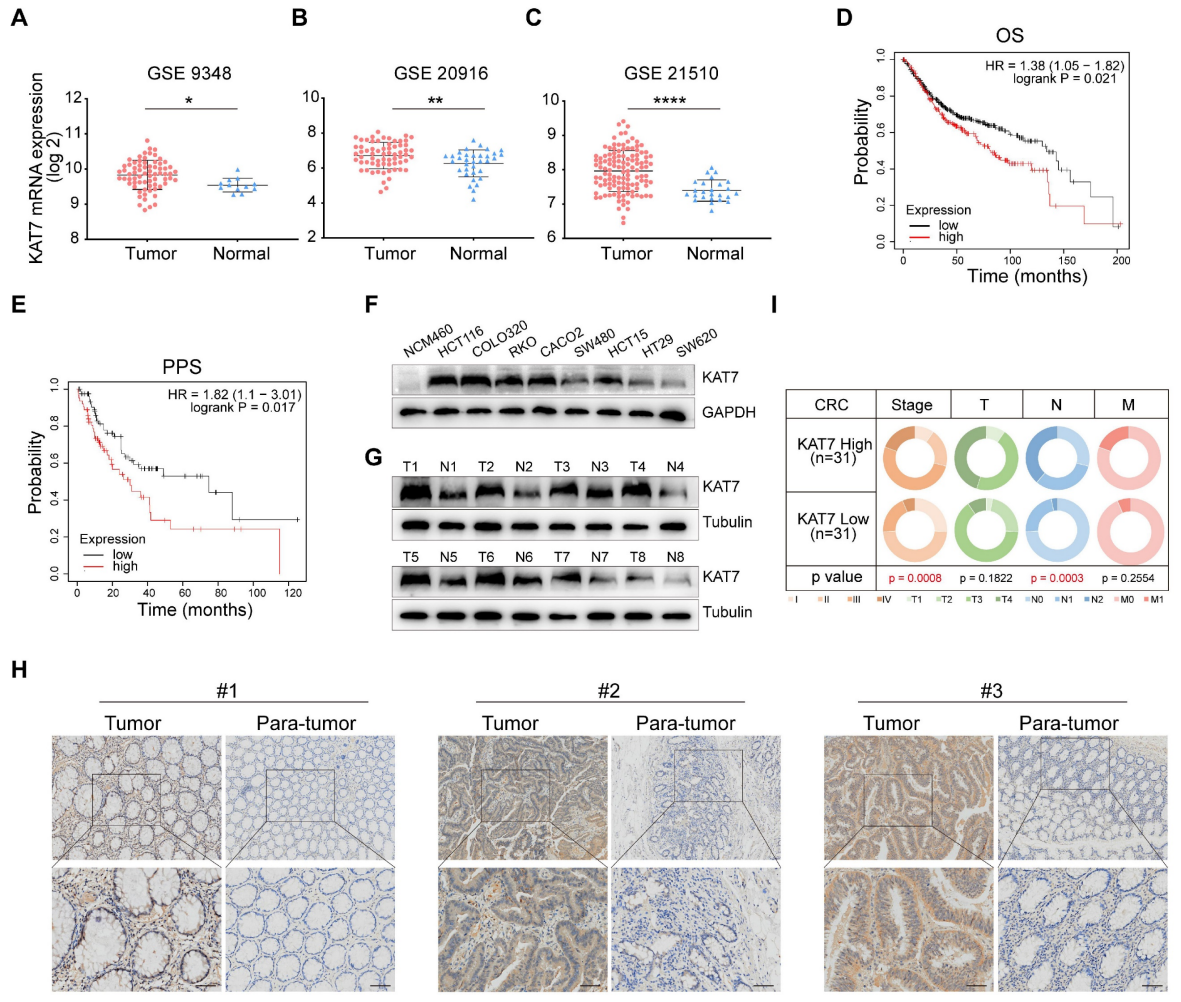
** KAT7 expression is significantly up-regulated in CRC. (A-C)** Expression levels of KAT7 mRNA in colorectal cancer (CRC) tissues and normal colorectal tissues were analyzed using data from the GSE9348 dataset 47 **(A)**, the GSE20916 dataset 48** (B)**, and the GSE21510 dataset 49 **(C)**. **(D-E)** Prognostic analysis of CRC patients with high/low expression of KAT7 was conducted using the Kaplan-Meier plotter database, evaluating overall survival (OS) **(D)** and post-progression survival (PPS) **(E)**. **(F)** The expression of the KAT7 protein was examined in normal colonic epithelial cells and various CRC cell lines. **(G)** KAT7 protein expression was determined in colorectal tumor tissues and adjacent normal tissues. **(H)** Immunohistochemical (IHC) analysis was performed to assess KAT7 expression in CRC tissues and adjacent normal tissues (Scale bar = 100 μm). **(I)** The TNM staging of CRC patients with high/low expression of KAT7 in our clinical samples. Error bars represent the mean ± standard deviation (SD). Statistical significance was determined using Two-tailed unpaired Student's t-tests **(A-C)** and Chi-square test** (I)**. *P < 0.05, **P < 0.01, ****P < 0.0001.

First, we investigated the expression levels of KAT7 in colorectal tumor samples and normal colorectal tissues by searching the GEO database. We noted that KAT7 levels were increased in CRC relative to normal controls (**Figure 1A-C**). To examine the prognostic value of KAT7 in individuals with CRC, we utilized the Kaplan-Meier plotter database to evaluate the correlation between survival time and different expression levels of KAT7. **Figure 1D-E** demonstrated a significant association between high levels of KAT7 and poor overall survival (OS) and post-progression survival (PPS) in CRC patients, respectively.

To validate these bioinformatics findings, we assessed the levels of protein expression for KAT7 in normal colonic epithelial cells (NCM460) and various CRC cell lines. The findings indicated that CRC cell lines showed markedly elevated KAT7 protein levels in contrast to normal colonic epithelial cells (**Figure 1F**). Western blot analysis (**Figure 1G**) showed increased KAT7 protein levels in CRC tissues from representative patients, whereas its expression was comparatively low in the adjacent normal colorectal tissues. A significant elevation of KAT7 expression in colorectal cancer compared to adjacent normal tissues was also demonstrated by immunohistochemical analysis (**Figure 1H**). Key genes involved in CRC progression are often associated with cancer stages. In our clinical sample data, CRC patients in the high-expression group of KAT7 showed more advanced stages and lymph node metastasis (**Figure 1I**). These results indicate that KAT7 is upregulated in CRC and correlates with poor survival outcomes.

### KAT7 knockdown suppresses cell viability, proliferation, and promotes apoptosis

**Figure 2:**
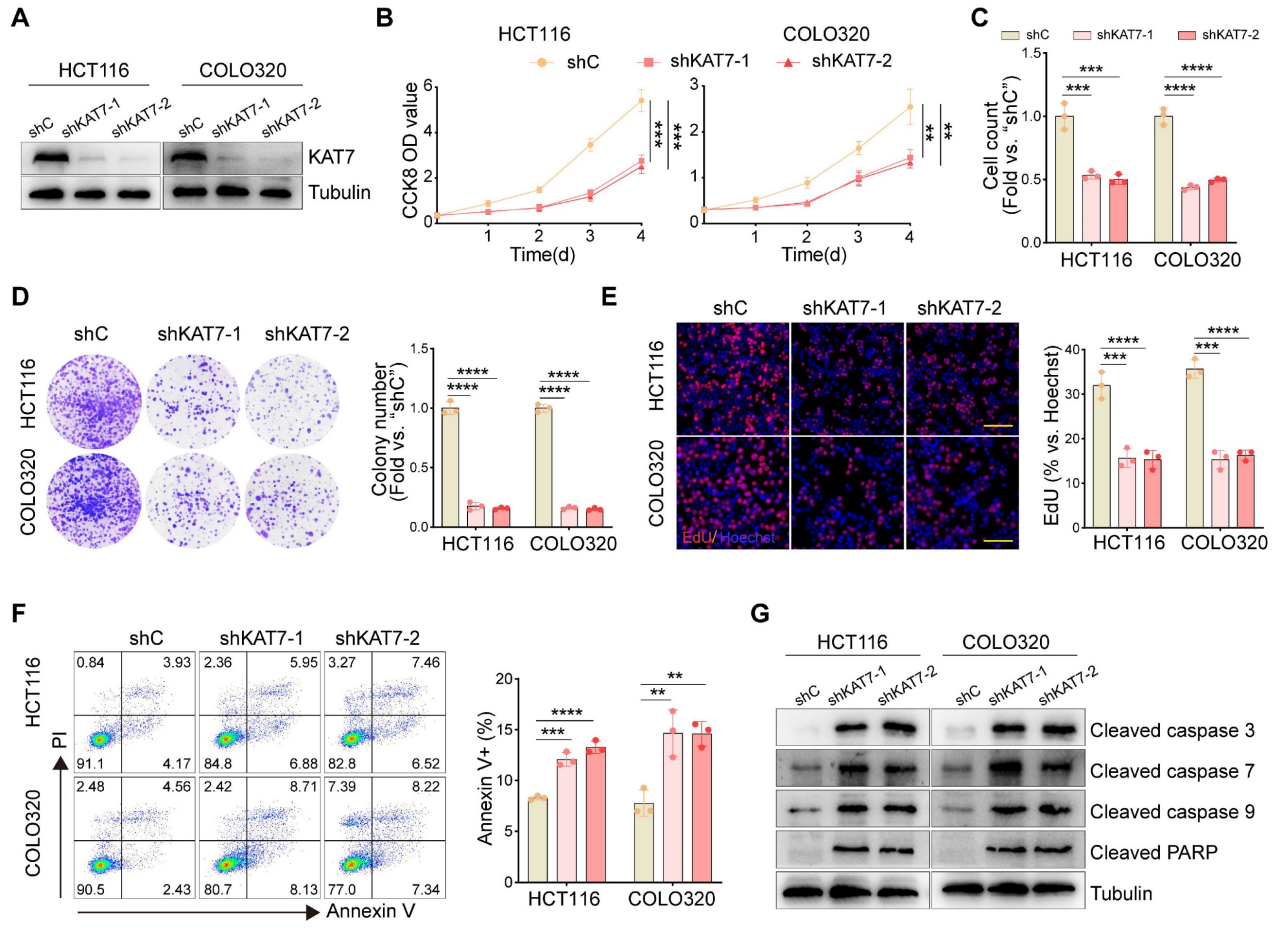
** KAT7 knockdown suppresses cell viability, proliferation, and promotes apoptosis. (A)** Western blot analysis was performed to assess the knockdown of KAT7 expression using shRNA.** (B)** CCK-8 assay was conducted to evaluate the viability of CRC cells after KAT7 knockdown. **(C)** CRC cells were treated as described in **(A)**, and cell proliferation was examined through cell counting. **(D)** The impact of KAT7 knockdown on the colony formation ability of cells was observed. **(E)** EdU assay was performed to investigate the effect of KAT7 knockdown on cell proliferation (left, Scale bar = 100 μm). The bar chart represents the percentage of EdU-positive cells (right). **(F)** Flow cytometry was employed to analyze apoptosis in CRC cells after KAT7 knockdown (left), and the percentage of apoptotic cells was quantified (right). **(G)** The expression of apoptosis-related proteins in CRC cells was treated as described in **(A)**. Error bars represent the mean ± SD. Statistical significance was determined using one-way ANOVA **(B-F)**. **P < 0.01, ***P < 0.001, ****P < 0.0001.

To examine the functional impact of KAT7 in CRC cells, two lentiviral shRNAs (shKAT7-1/shKAT7-2) specifically targeting distinct regions of human KAT7, along with a puromycin selection gene, were individually transfected into HCT116 and COLO320 cells, which exhibited the highest expression levels of KAT7 (**Figure 1F**). Western blot analysis confirmed a significant downregulation of KAT7 expression (**Figure 2A**). Compared to cells transfected with shC, KAT7 knockdown cells exhibited a remarkable decrease in viability (**Figure 2B**). The proliferation of KAT7 knockdown cells was substantially hindered, as demonstrated by the results in **Figure 2C**. Moreover, KAT7 knockdown markedly reduced colony formation (**Figure 2D**) and nuclear EdU incorporation (**Figure 2E**), further confirming the suppression of proliferation.

The impact of KAT7 shRNA on cell apoptosis was subsequently examined. Following KAT7 shRNA transfection, a notable increase in Annexin V-positive cells was observed (**Figure 2F**). Additionally, increased cleavage of caspase-3, caspase-7, caspase-9, and PARP (poly ADP-ribose polymerase) was observed (**Figure 2G**). These results collectively indicate that silencing of KAT7 can impede cell viability and proliferation while inducing apoptosis in CRC cells.

### KAT7 silencing impairs migration and invasion in CRC cells

**Figure 3:**
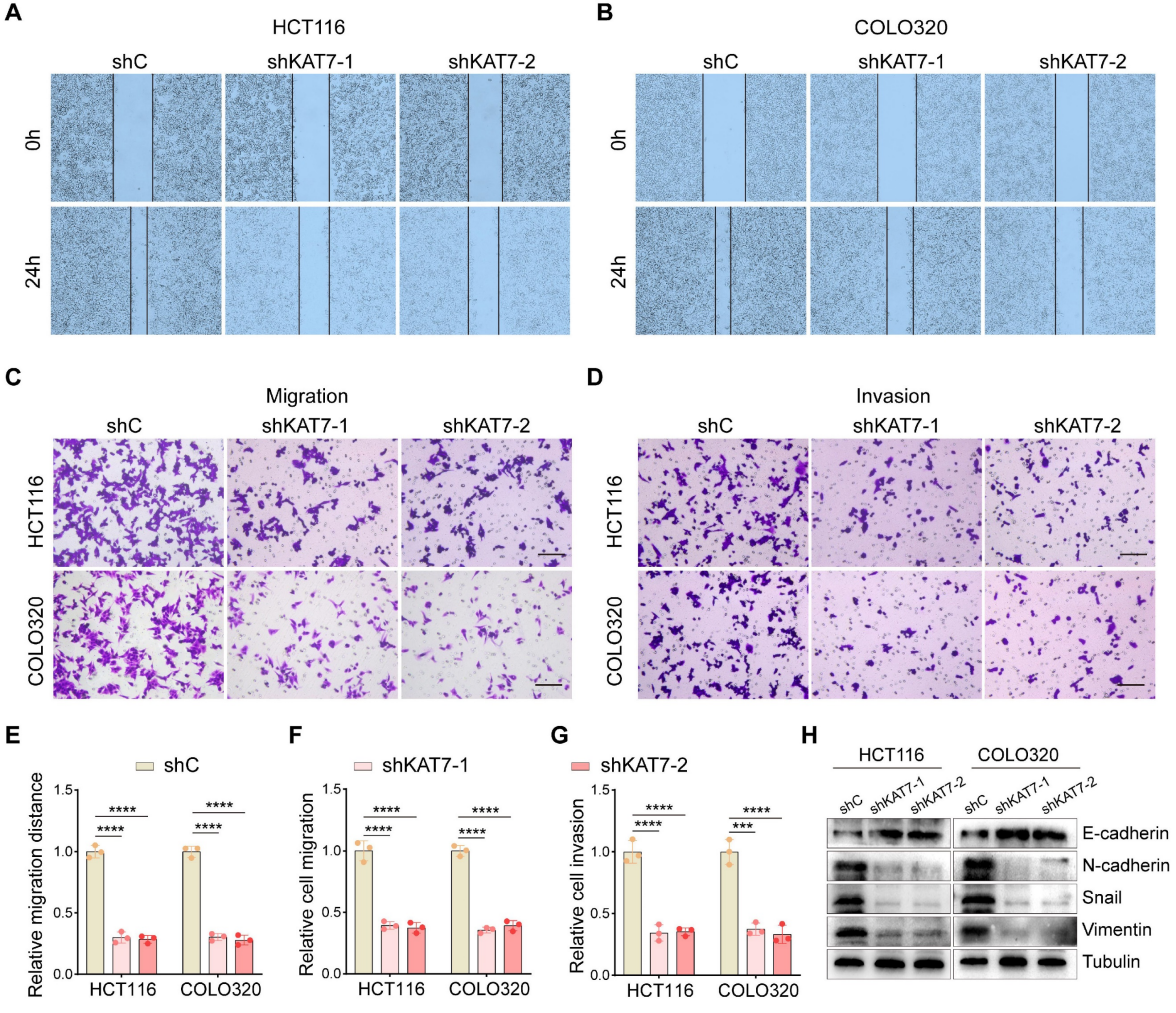
** KAT7 silencing impairs migration and invasion in CRC cells. (A-B)** Wound-healing experiments were conducted to observe cell migration after shRNA-mediated knockdown of KAT7. **(C-D)** The impact of KAT7 knockdown on the migration **(C)** and invasion** (D)** abilities of CRC cells was assessed (Scale bar = 100 μm). **(E)** Statistical analysis of cell migration distance in **(A)** and **(B)**. **(F)** Statistical analysis of cell migration quantity in **(C)**. **(G)** Statistical analysis of cell invasion quantity in **(D)**. **(H)** The effect of KAT7 knockdown on the expression of proteins associated with epithelial-mesenchymal transition (EMT). Error bars represent the mean ± SD. Statistical significance was assessed using one-way ANOVA **(E-G)**. ***P < 0.001, ****P < 0.0001.

Given the critical role of increased motility in tumor metastasis 33,34, wound-healing assays were performed *in vitro* to evaluate the impact of KAT7 silencing on CRC cell behavior. The results revealed that KAT7 knockdown significantly impeded the migration of HCT116 and COLO320 cells (**Figure 3A-B, E**). Furthermore, transwell chamber assays demonstrated a significant decrease in cell migration and invasion upon KAT7 downregulation in CRC cells (**Figure 3C-D, F-G**). Epithelial-mesenchymal transition (EMT) is a pivotal process that influences tumor cells' migratory and invasive properties 35. We observed that increased levels of E-cadherin and decreased levels of N-cadherin, Snail, and Vimentin resulted from reduced KAT7 expression, consistently (**Figure 3H**). Collectively, these findings establish that KAT7 silencing in CRC cells profoundly affects their migration and invasion capabilities.

### CRISPR/Cas9-induced KAT7 knockout suppresses CRC cell activity

To eliminate the possibility of off-target effects caused by KAT7 shRNA, we employed CRISPR/Cas9 to knockout KAT7 in HCT116 and COLO320 cells (**Figure S1A**). Functional studies further showed that CRISPR/Cas9-mediated KAT7 knockout significantly decreased cell viability (**Figure S1B**), proliferation (**Figure S1C**), and colony formation (**Figure S1D**). The flow cytometric analysis revealed an increase in Annexin V^+^ cells upon KAT7 knockout (**Figure S1E-F**). Furthermore, the levels of apoptosis-related proteins, such as cleaved caspase-3, caspase-7, caspase-9, and PARP, were increased (**Figure S1G**). These findings indicate that the knockout of KAT7 activates apoptosis in CRC cells.

Furthermore, we investigated the impact of KAT7 knockout on CRC cell migration, invasion, and EMT. Wound-healing assays revealed a substantial decrease in the migration speed of CRC cells following KAT7 knockout (**Figure S2A-B, E**). Transwell chamber experiments also revealed a notable decrease in cell migration (**Figure S2C, F**) and invasion (**Figure S2D, G**) abilities upon KAT7 knockout. Additionally, we examined the expression of EMT-related proteins and found that the knockout of KAT7 led to a significant upregulation of E-cadherin expression, while the expression of N-cadherin, Snail, and Vimentin was downregulated (**Figure S2H**). These findings suggest that the migratory and invasive capabilities of CRC cells are significantly inhibited upon KAT7 knockout, which is consistent with the findings obtained from KAT7 knockdown using shRNA. In summary, CRISPR/Cas9-mediated KAT7 knockout demonstrated significant anti-CRC cell activity.

### KAT7 overexpression drives oncogenic progression in CRC cells

**Figure 4:**
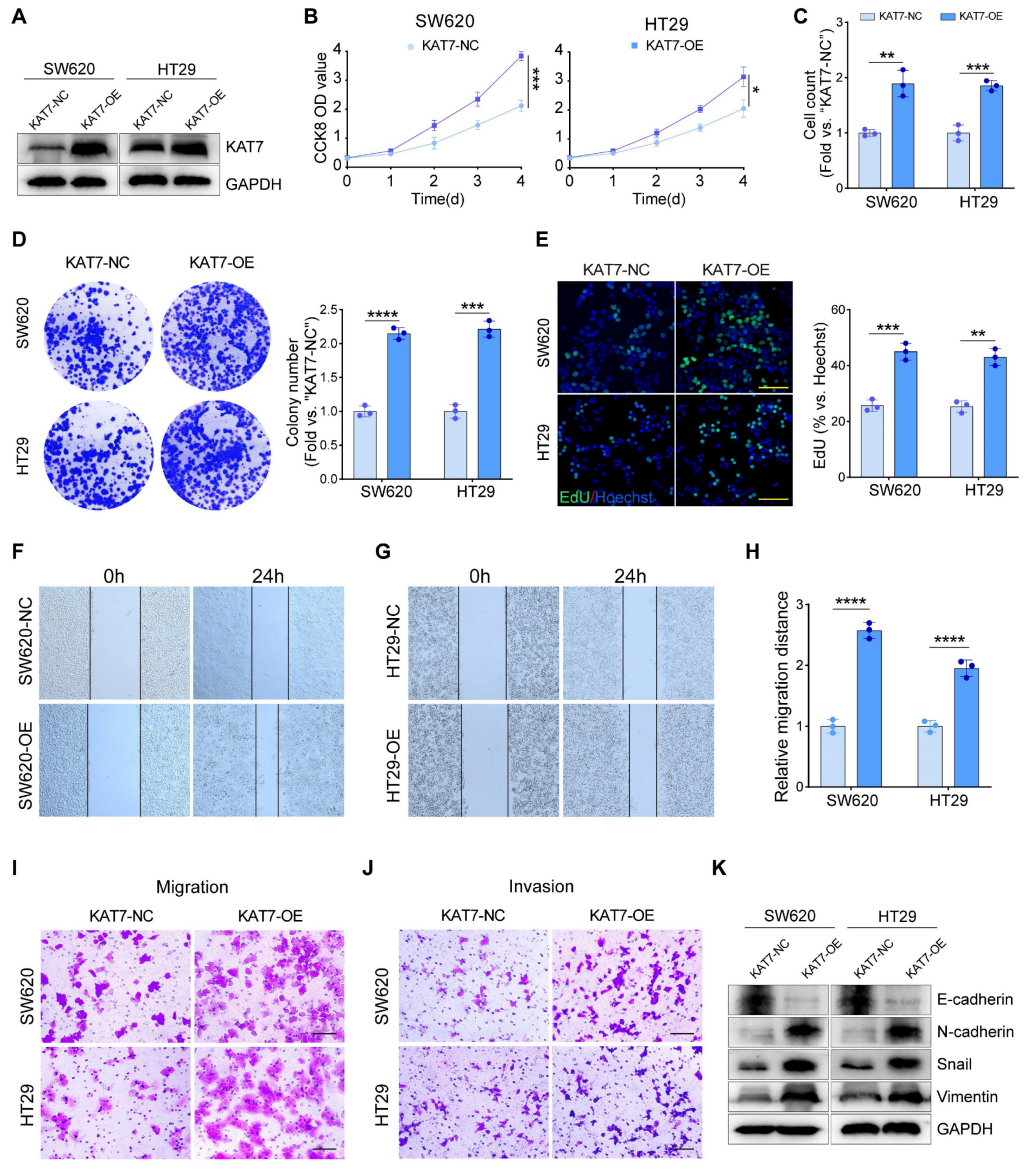
** KAT7 overexpression drives oncogenic progression in CRC cells. (A)** Overexpression of KAT7 was performed in SW620 and HT29 cells. **(B)** Impact of KAT7 overexpression on CRC cell viability. **(C)** Cell counting assessment of CRC cell proliferation after KAT7 overexpression.** (D)** Colony formation assay after KAT7 overexpression (left) and corresponding colony count (right).** (E)** EdU experiment evaluating the proliferation of CRC cells treated as in **(A)**, fluorescence image (left, Scale bar = 100 μm), and a statistical bar graph (right). **(F-H)** Changes in migration ability of SW620 **(F)** and HT29 **(G)** cells after KAT7 overexpression, and migration distance statistics **(H)**. **(I)** Transwell chamber experiment observing the effect of KAT7 overexpression on cell migration ability (Scale bar = 100 μm). **(J)** Enhanced invasion ability of CRC cells after KAT7 overexpression (Scale bar = 100 μm). **(K)** Expression changes of EMT-related proteins in CRC cells after being treated as in** (A)**. Error bars represent the mean ± SD. Statistical significance was determined using Two-tailed unpaired Student's t-tests **(B-E, H)**. *P < 0.05, **P < 0.01, ***P < 0.001, ****P < 0.0001.

Considering the suppressive impact of KAT7 silencing on CRC cell proliferation, migration, and invasion, we proposed that ectopic overexpression of KAT7 might yield the opposite outcome. To investigate this hypothesis, we transfected SW620 and HT29 cells with a construct designed to overexpress KAT7 (KAT7-OE), which have relatively low endogenous KAT7 levels (**Figure 1F**). Stable KAT7-OE cell lines were established through blasticidin selection. Western blot analysis confirmed a significant upregulation of KAT7 expression upon transfection with the KAT7 overexpression virus (**Figure 4A**).

We found that KAT7 overexpression substantially enhanced the viability (**Figure 4B**), proliferation (**Figure 4C**), colony formation (**Figure 4D**), and nuclear EdU incorporation (**Figure 4E**) of CRC cells. Wound-healing assays demonstrated an accelerated migration rate of CRC cells following KAT7 overexpression (**Figure 4F-H**). Moreover, transwell chamber assays demonstrated a marked enhancement in the migratory and invasive abilities of CRC cells following KAT7 overexpression (**Figure 4I-J**). Finally, we assessed the levels of EMT-related proteins via western blot analysis. We detected a significant decrease in E-cadherin and an increase in N-cadherin, Snail, and Vimentin levels in SW620 and HT29 cells upon KAT7 overexpression (**Figure 4K**), indicating the promotion of EMT in CRC cells. Overall, our findings suggest that overexpression of KAT7 has the potential to accelerate the progression of colorectal cancer *in vitro*.

### KAT7 knockdown attenuates MAPK/ERK signaling pathway activity

**Figure 5:**
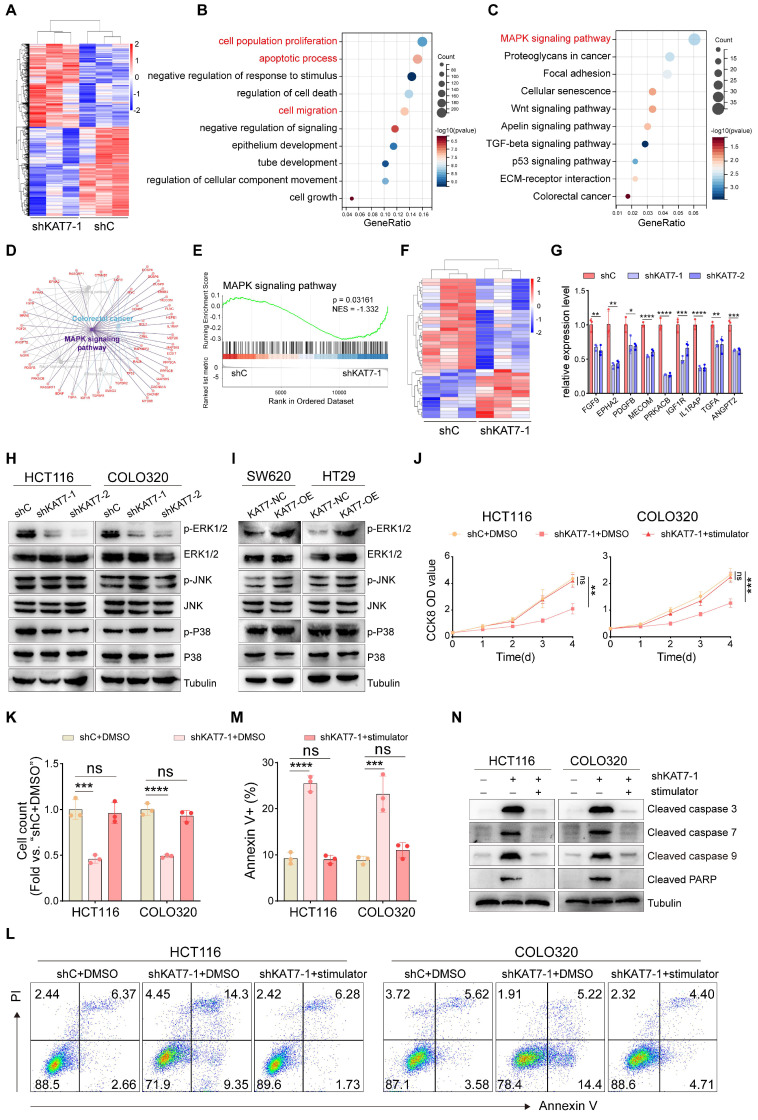
** KAT7 knockdown attenuates MAPK/ERK signaling pathway activity. (A)** Heatmap of differentially expressed genes in KAT7-knockdown and control HCT116 cells generated using R software. **(B)** Functional categorization based on gene ontology (GO) term enrichment. **(C)** Signaling pathway analysis based on KEGG enrichment of subcategories in signal transduction pathways. **(D)** Functionally grouped networks based on Kyoto Encyclopedia of Genes and Genomes (KEGG) pathway analysis, showing genes in KAT7-knockdown and control cells. **(E)** Gene set enrichment analysis (GSEA) of the MAPK signaling pathway in HCT116 cells treated as described in **(A)**. **(F)** Heatmap of differentially expressed genes related to the MAPK signaling pathway in HCT116 control and KAT7 knockdown cells, analyzed using R software. **(G)** Quantitative real-time PCR analysis of relative mRNA expression of indicated genes in control and KAT7-knockdown HCT116 cells. **(H)** Impact of KAT7 knockdown on the expression of proteins related to the MAPK pathway. **(I)** Detection of MAPK pathway-related protein expression levels after KAT7 overexpression. **(J)** Cell viability of CRC cells with or without KAT7 knockdown following 8 hours of treatment with 0.1% DMSO or the MAPK/ERK agonist C6 Ceramide (5 μM). **(K)** Cell proliferation measurement in cells treated as described in **(J)**. **(L-M)** Flow cytometry scatter plot **(L)** and a bar graph **(M)** showing apoptosis analysis of CRC cells treated as described in** (J)**. **(N)** Impact of treatment described in **(J)** on the expression of apoptosis-related proteins in CRC cells. Error bars represent the mean ± SD. Statistical significance was assessed using one-way ANOVA **(G, J-K, M)**. *P < 0.05, **P < 0.01, ***P < 0.001, ****P < 0.0001. ns indicates no significance.

Subsequently, we performed RNA-seq analysis to investigate the molecular pathways through which KAT7 facilitates the progression of CRC. We identified a total of 1708 differentially expressed genes in KAT7 silenced HCT116 cells in comparison to the control group (**Figure 5A**). In alignment with the findings from the phenotype analysis, gene ontology (GO) term enrichment analysis revealed significant enrichment in cell proliferation, apoptotic process, and migration functions (**Figure 5B**). Kyoto Encyclopedia of Genes and Genomes (KEGG) analysis revealed enrichment of the MAPK signaling pathway (**Figure 5C**). To achieve a more comprehensive understanding of the functionally grouped networks involved in CRC, we employed the ClueGO and CluePedia plugins in Cytoscape. Using these tools, we were able to examine the functionally grouped network between the KAT7 knockdown and control groups and identify pathways enriched in tumorigenesis. Our findings revealed a strong relationship between the MAPK pathway and CRC (**Figure 5D**). Gene set enrichment analysis (GSEA) indicated significant suppression of MAPK signaling pathways following KAT7 knockdown (**Figure 5E**). Consistently, the heatmap showcased distinct gene expression profiles related to the MAPK pathway (**Figure 5F**). Furthermore, qRT-PCR analysis of 9 crucial genes involved in tumor progression, consistent with the heatmap data, demonstrated their downregulation upon KAT7 knockdown (**Figure 5G**).

The MAPK signaling pathway comprises three families: ERK, P38, and JNK. Next, we confirmed the influence of KAT7 on the MAPK signaling pathway through western blot experiments. Knockdown of KAT7 reduced the phosphorylation of ERK1/2, while KAT7 overexpression significantly increased their phosphorylation. However, no significant changes were observed in the phosphorylation of JNK and P38, nor the total levels of ERK1/2, JNK, and P38 (**Figure 5H-I**). *In vitro* experiments demonstrated that activation of the MAPK/ERK cascade can rescue the anti-tumor effect induced by KAT7 knockdown, as evidenced by the restoration of cell viability and proliferation (**Figure 5J-K**), as well as the suppression of apoptosis activation (**Figure 5L-N**). Collectively, these findings suggest that KAT7 promotes CRC development through the activation of the MAPK/ERK signaling pathway.

### KAT7 upregulates MRAS expression to activate the MAPK/ERK signaling pathway in CRC cells

**Figure 6:**
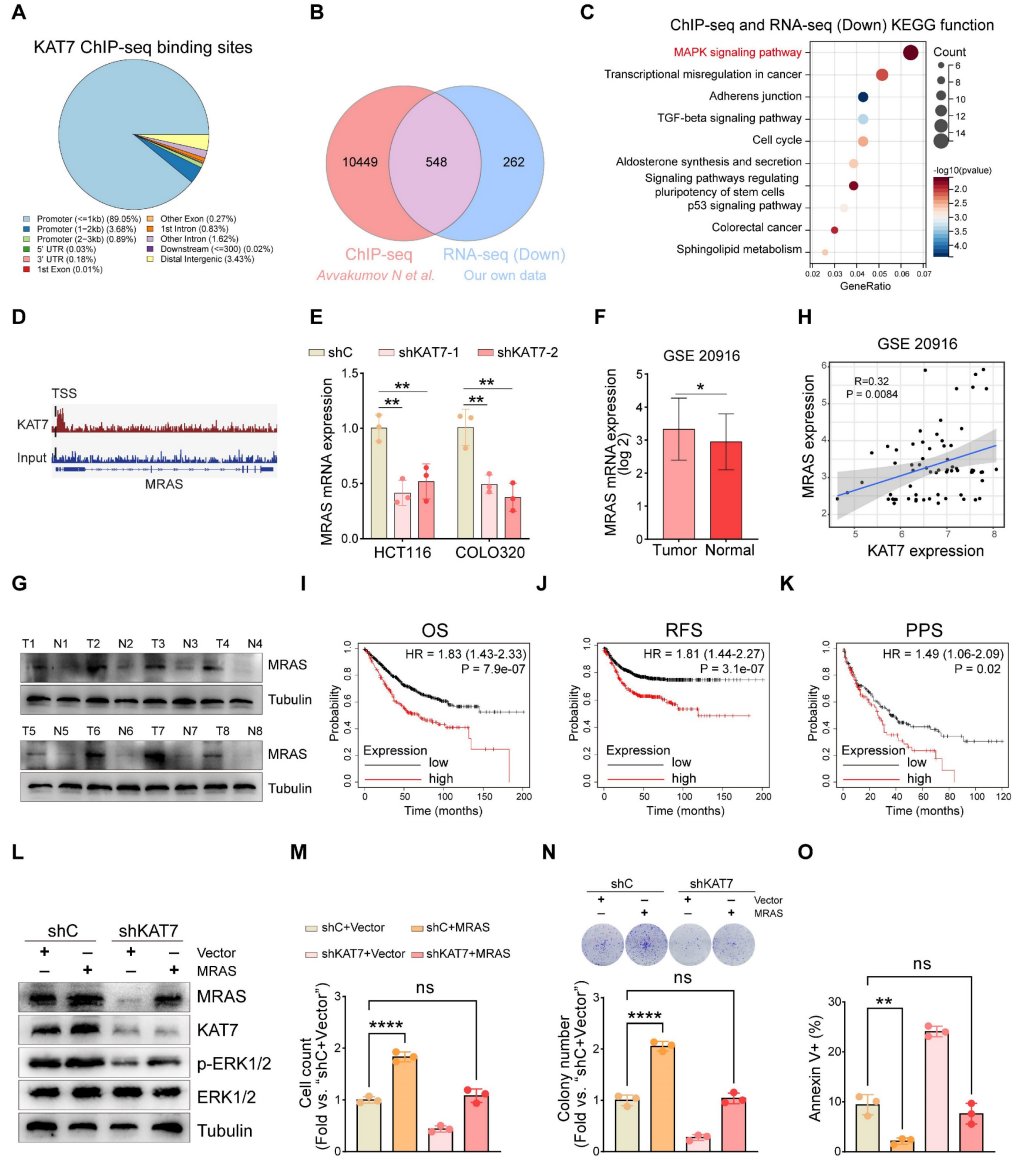
** KAT7 upregulates MRAS expression to activate the MAPK/ERK signaling pathway in CRC cells. (A)** Pie chart displaying the distribution of KAT7 binding sites on genes in ChIP-seq, with data obtained from a public database. **(B)** Venn diagram showing the overlap between genes bound by KAT7 in ChIP-seq and genes downregulated in RNA-seq after KAT7 knockdown. **(C)** KEGG analysis reveals the genes overlapping between ChIP-seq and RNA-seq as shown in **(B)**. **(D)** Peak plot illustrating the binding of KAT7 in the promoter region of the MRAS gene. **(E)** qRT-PCR measuring the impact of KAT7 knockdown on MRAS gene expression. **(F)** Expression profile of the MRAS gene in CRC tissues and normal individuals, with data obtained from a public database 48. **(G)** Expression of the MRAS protein in CRC tissues and adjacent normal tissues. **(H)** Correlation analysis between MRAS mRNA and KAT7 mRNA expression, with data obtained from a public database 48. **(I-K)** Kaplan-Meier plotter database analysis of the relationship between MRAS expression and prognosis in CRC patients, including OS **(I)**, recurrence-free survival (RFS)** (J)**, and PPS **(K)**. **(L)** Expression profile of indicated proteins in KAT7-knockdown/untargeted HCT116 cells overexpressing/not overexpressing MRAS. **(M-O)** Effects on cell proliferation **(M)**, colony formation **(N)**, and apoptosis** (O)** in HCT116 cells treated as described in **(L)**. Error bars represent the mean ± SD. Statistical significance was assessed using one-way ANOVA** (E, M-O)**, Two-tailed unpaired Student's t-tests** (F),** and nonlinear regression analysis **(H)**. *P < 0.05, **P < 0.01, ****P < 0.0001. ns indicates no significance. TSS: Transcription start site.

KAT7, as an epigenetic regulator, has been shown to modulate gene transcription by promoting histone acetylation. To clarify the precise mechanism by which KAT7 regulates the MAPK/ERK signaling pathway, we retrieved the publicly available KAT7 ChIP-Seq dataset of CRC 36. A total of 10,997 filtered peaks were detected, predominantly situated within the promoter regions of target genes (**Figure 6A**). By performing a combined analysis of RNA-seq and ChIP-seq data of KAT7, we found that 548 genes downregulated in RNA sequencing overlapped with the genes identified in ChIP-seq (**Figure 6B**). KEGG functional enrichment analysis of the overlapping genes highlighted a significant concentration in the MAPK signaling pathway (**Figure 6C**).

Considering that MAPK/ERK is a widely recognized pathway with protumorigenic effects in colorectal tumorigenesis 37, our subsequent objective was to pinpoint the specific upstream components of MAPK influenced by KAT7. The conventional MAPK/ERK signaling cascade involves a sequential activation of EGFR, RAS, and RAF proteins, leading to an amplification of their signaling through MEK phosphorylation and, ultimately, ERK activation 38,39. MRAS is a member of the RAS family. It forms a complex with SHOC2 and PP1C to dephosphorylate RAF family proteins at crucial inhibitory phosphorylation sites, leading to the activation of the MAPK/ERK pathway 40-42. Furthermore, acetylated histones in promoter regions are essential for the regulation of gene transcription. Based on this, we hypothesized that KAT7 may influence the activity of the MAPK/ERK signaling pathway by regulating the expression of the MRAS gene. Through the analysis of ChIP-seq data, we have observed that KAT7 directly binds to the MRAS locus and predominantly localizes downstream of the transcription start site (TSS) of MRAS (**Figure 6D**). These findings suggest that KAT7 may exert an influence on the transcription of the MRAS gene. Our RNA-seq data revealed that the knockdown of KAT7 resulted in downregulation of MRAS mRNA expression (data not shown), which was further confirmed by qRT-PCR results (**Figure 6E**). Furthermore, we investigated MRAS expression in CRC and its correlation with patient prognosis. We discovered that MRAS expression was higher in colorectal tumors compared to normal colorectal tissue (**Figure 6F**). Western blot experiments demonstrated significantly higher levels of MRAS protein expression in CRC tumor tissue relative to adjacent normal tissue (**Figure 6G**). Next, we investigated the relationship between MRAS and KAT7 mRNA expression. The findings revealed a positive association between MRAS and KAT7 expression levels (**Figure 6H**). Survival analysis using the Kaplan-Meier plotter database indicated that CRC patients with high MRAS expression had poor OS, relapse-free survival (RFS), and PPS outcomes (**Figure 6I-K**).

To investigate whether overexpression of MRAS could rescue the functional defects caused by KAT7 knockdown in CRC cells, we performed rescue experiments. Western blot analysis confirmed successful overexpression of MRAS in HCT116-shC and HCT116-shKAT7 cells, without affecting the endogenous expression of KAT7. Additionally, the overexpression of MRAS reversed the reduced phosphorylation of ERK1/2 resulting from KAT7 knockdown, signifying the reactivation of the MAPK/ERK signaling pathway (**Figure 6L**). Functional experiments demonstrated that overexpression of MRAS increased the proliferation and colony-forming capability of HCT116-shC cells while reducing apoptosis. Moreover, it successfully restored the decreased proliferation and colony formation ability and activated apoptosis caused by KAT7 knockdown in HCT116 cells (**Figure 6M-O**).

### KAT7 acetyltransferase activity is required for activating MAPK/ERK signaling

**Figure 7:**
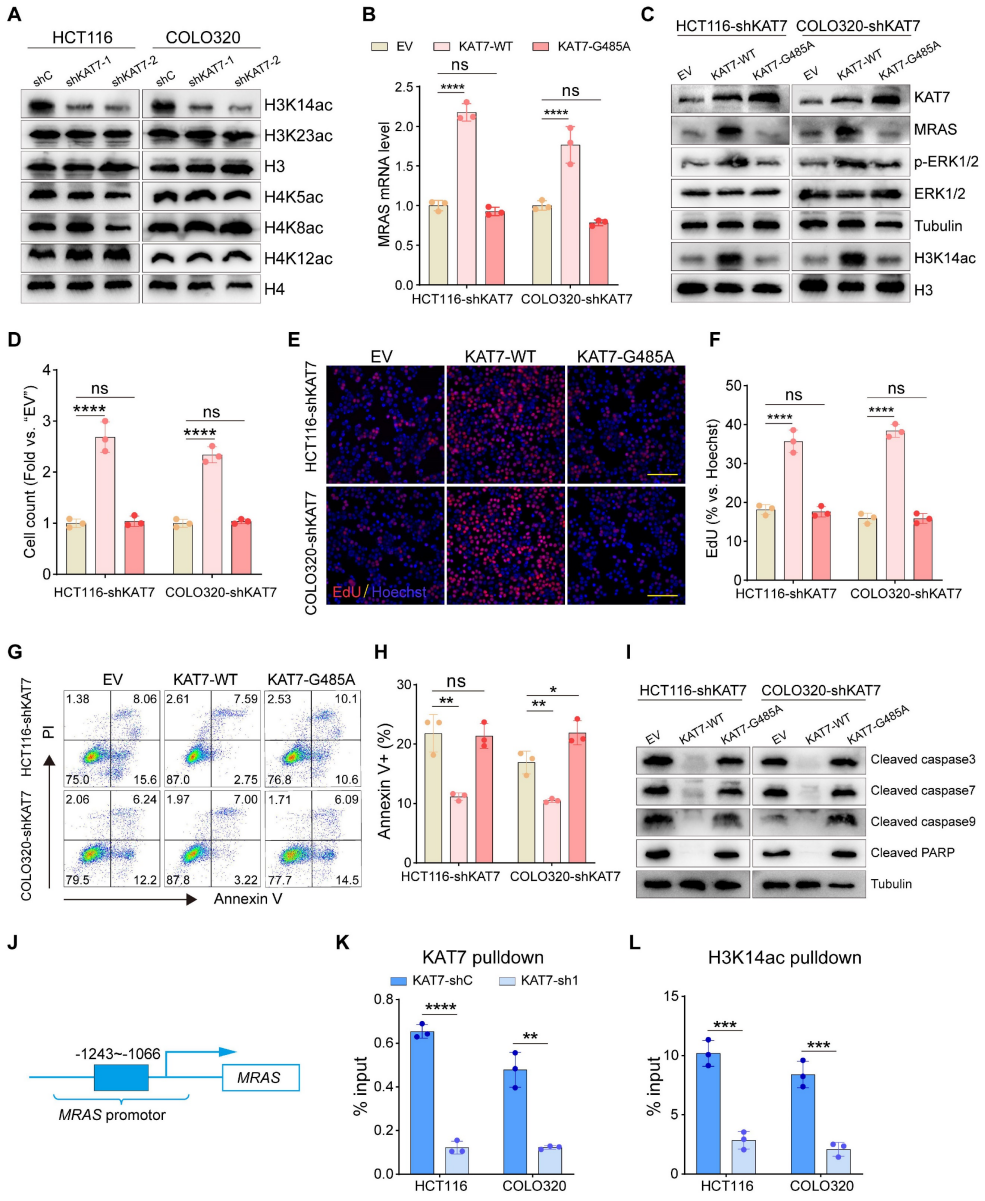
** KAT7 acetyltransferase activity is required for activating MAPK/ERK signaling. (A)** Impact of KAT7 knockdown on histone acetylation. **(B)** Expression changes of the MRAS gene in KAT7-knockdown CRC cells overexpressing wild-type KAT7 (KAT7-WT) or acetylation-deficient KAT7 mutant (KAT7-G485A). **(C)** Western blot analysis of protein expression in HCT116/COLO320-shKAT7 cells treated as described in **(B)**. **(D-I)** Cell proliferation measurement **(D-F)**, flow cytometry analysis of cell apoptosis **(G-H)**, and expression changes of apoptosis-related proteins **(I)** in HCT116/COLO320-shKAT7 cells overexpressing KAT7-WT or KAT7-G485A. Scale bar = 100 μm** (E)**. **(J-K)** ChIP-qPCR assays were conducted on CRC cells treated with either control shRNA or KAT7 shRNA. Chromatin DNA was immunoprecipitated using antibodies specific for KAT7 or acetylated H3K14. The results showed that KAT7 knockdown reduced the binding of KAT7 (**K**) and the acetylation of H3K14 (**L**) on the promoter region (-1243~-1066) of the MRAS gene (**J**). Error bars represent the mean ± SD. Statistical significance was evaluated using one-way ANOVA for experiments (**B, D, F, H**) and two-tailed unpaired Student's t-tests (**K-L**). *P < 0.05, **P < 0.01, ***P < 0.001, **P < 0.0001. ns indicates no significance.

KAT7 is known to assemble into two distinct HAT complexes, each targeting the acetylation of either histone H3 or H4 24,43. Thus, we investigated the acetylation levels of H3 and H4 tails in KAT7 knockdown HCT116 and COLO320 cells. Among the possible acetylation sites targeted by KAT7, we noted a substantial decrease in the acetylation of the Lysine-14 residue of H3 (H3K14ac) upon KAT7 knockdown, while the acetylation of other sites remained unaffected (**Figure 7A**).

To explore whether the acetyltransferase function of KAT7 is crucial for activating MAPK/ERK signaling, we generated a mutant variant, KAT7-G485A, with deficient acetyltransferase activity 44. Our qRT-PCR experiments demonstrated a significant increase in MRAS mRNA expression following the reintroduction of wild-type KAT7 (KAT7-WT) in CRC cells with KAT7 knockdown (**Figure 7B**). Additionally, western blot analysis revealed elevated levels of H3K14ac, MRAS, and p-ERK1/2 proteins upon the overexpression of KAT7-WT (**Figure 7C**). These findings indicate the reactivation of the MAPK/ERK pathway upon the reintroduction of KAT7-WT in KAT7 knockdown CRC cells. Functional experiments demonstrated that KAT7-WT overexpression significantly promoted the proliferation of KAT7 knockdown cells (**Figure 7D-F**) and reduced cell apoptosis (**Figure 7G-I**) compared to the empty vector (EV) group. In contrast, the overexpression of the KAT7-G485A mutant did not affect MRAS expression, H3K14 acetylation, phosphorylation of ERK1/2 (**Figure 7B-C**), cell proliferation (**Figure 7D-F**), or apoptosis (**Figure 7G-I**) in KAT7 knockdown CRC cells.

The presence of acetylated histones within promoter regions is crucial for the regulation of gene transcription 43. To examine KAT7's binding to the MRAS promoter region, we performed a ChIP-qPCR assay using KAT7 antibody. Our findings indicated that KAT7 associates with the MRAS promoter region, and this interaction was weakened in KAT7 knockdown CRC cells (**Figure 7J-K**). Additionally, **Figure 7A** shows that H3K14 acetylation in CRC cells depends on KAT7 expression. Using H3K14ac antibodies, we conducted ChIP-qPCR assays that revealed enrichment of H3K14ac in the MRAS promoter region. Similar to KAT7, the binding of H3K14ac was significantly reduced in KAT7 knockdown CRC cells (**Figure 7L**).

Since our results indicate that the HAT activity of KAT7 is critical for the survival of CRC cells and the activation of the MAPK/ERK signaling pathway, we next utilized a small molecule inhibitor, WM-3835, which significantly suppresses the HAT activity of KAT7 21, to investigate its potential anti-CRC effects. Cell viability assays demonstrated that WM-3835 effectively inhibited the viability of CRC cells without affecting the viability of normal intestinal epithelial cells (NCM460) (**Figure S3A**). We further found that KAT7 knockdown markedly attenuated the inhibitory effects of WM-3835 (**Figure S3B**). These results suggest that WM-3835 exhibits good safety and functions in CRC by targeting KAT7. Additionally, WM-3835 inhibited the proliferation of CRC cells (**Figure S3C-D**) while promoting apoptosis (**Figure S3E-F**). Moreover, similar to the effects observed with KAT7 knockdown, WM-3835 significantly suppressed H3K14 acetylation (**Figure S3G**), transcription of the *MRAS* gene (**Figure S3H**), and activation of MAPK/ERK signaling (**Figure S3I**). These findings suggest that KAT7 facilitates MRAS transcription by acetylating H3K14, thereby playing a crucial role in the activation of MAPK/ERK signaling.

### Knockdown of KAT7 inhibits CRC growth and metastasis *in vivo*

**Figure 8:**
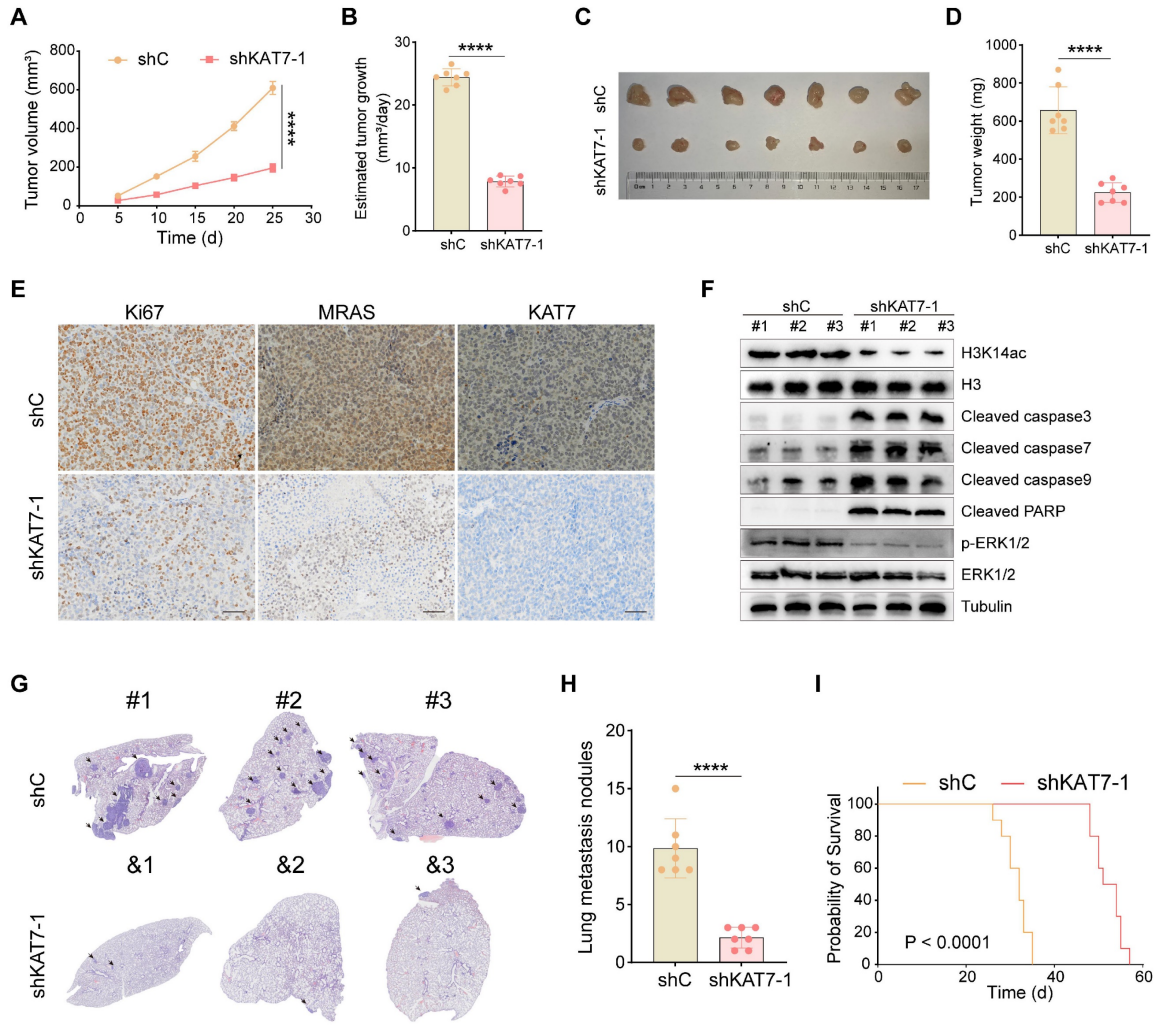
** Knockdown of KAT7 inhibits CRC growth and metastasis *in vivo*. (A)** Observation of subcutaneous tumor growth rate in COLO320 cells after KAT7 knockdown (n = 7 per group). **(B)** Evaluation of average tumor growth rate 25 days after tumor cell inoculation. **(C)** Collection and photography of subcutaneous tumors 25 days after tumor cell inoculation. **(D)** Bar graph depicting the statistical analysis of subcutaneous tumor mass. **(E)** Immunohistochemical detection of Ki67, MRAS, and KAT7 expression in subcutaneous tumors after KAT7 knockdown (Scale bar = 100 μm). **(F)** Western blot analysis of protein expression in subcutaneous tumors after KAT7 knockdown. **(G)** Assessment of CRC lung metastasis in B-NDG mice through tail vein injection of COLO320 cells with or without KAT7 knockdown (n = 7 per group). **(H)** Statistical analysis of the number of lung metastatic tumors in** (G)**. **(I)** Impact of KAT7 knockdown on the survival of CRC lung metastasis mice (n = 10 per group). Error bars represent the mean ± SD. Statistical significance was assessed using two-tailed unpaired Student's t-tests for experiments **(A-B, D, H)** and log-rank test for experiment** (I)**. ****P < 0.0001.

Finally, we examined the possible effects of KAT7 on CRC growth and metastasis *in vivo*. COLO320 cells with shKAT7-1 or shC were inoculated via subcutaneous injection into B-NDG mice. The results demonstrated that shKAT7-1 COLO320 xenografts exhibited significantly slower growth compared to the control xenografts (**Figure 8A**). Calculating the average daily tumor growth using the formula (Tumor volume on Day 25)/25 revealed that KAT7 knockdown inhibited COLO320 xenograft growth *in vivo* (**Figure 8B**). All tumors were individually weighed on Day 25, revealing that the tumors in the shKAT7-1 group weighed significantly less than those in the shC group (**Figure 8C-D**).

Furthermore, immunohistochemical analysis was performed on the tumors. Compared to the control group, KAT7 knockdown led to a marked decrease in the expression of Ki67 and MRAS (**Figure 8E**). Western blot analysis of the tumors showed a significant reduction in H3K14ac and p-ERK1/2 expression in the shKAT7 group, while the expression of cleaved caspase3/7/9/PARP was significantly upregulated, aligning with the earlier *in vitro* experimental findings (**Figure 8F**).

Our previous *in vitro* experiments demonstrated that KAT7 knockdown could substantially hinder the migration and invasion of CRC cells (**Figure 3**). Next, we investigated whether the same effect could be observed in mice. We injected COLO320 cells, either with KAT7 knockdown or without, into the mice via the tail vein. The research findings showed that KAT7 knockdown significantly suppressed the lung metastasis of CRC (**Figure 8G-H**). Finally, survival analysis results demonstrated that knocking down KAT7 in COLO320 cells noticeably extended the survival time of the tumor-bearing mice (**Figure 8I**). The above results indicate that knocking down KAT7 in CRC cells can inhibit their growth and metastasis *in vivo*.

## Discussion

In this study, we described a novel mechanism in which KAT7 upregulates the MAPK/ERK signaling pathway, playing a crucial role in CRC progression. KAT7 promotes H3K14 acetylation, resulting in increased expression of MRAS and activation of the MAPK/ERK pathway, ultimately leading to enhanced CRC tumorigenesis.

Our findings demonstrate that KAT7 functions as an oncogene in CRC. Previous research has shown that KAT7 is essential for maintaining leukemia stem cells through the acetylation of histone H3 at K14 21. Additionally, another study revealed the overexpression of KAT7 in osteosarcoma, where it functions as a newly identified oncogenic gene crucial for tumor development and progression. This study also identified ZNF384 as a key transcription factor that directly associates with the KAT7 promoter 45. In our study, we observed upregulated expression of KAT7 in CRC samples. Furthermore, analysis of CRC patient samples revealed an inverse relationship between KAT7 expression and patient survival. shRNA-mediated knockdown of KAT7 inhibited CRC cell growth, migration, and invasion while enhancing apoptosis. Furthermore, CRISPR/Cas9-mediated knockout of KAT7 also produced consistent outcomes. Conversely, overexpression of KAT7 significantly enhanced CRC cell proliferation, migration, and invasion abilities. These findings highlight the critical role of KAT7 in CRC tumorigenesis.

Moreover, our findings suggest that KAT7 mediates CRC progression through the MAPK/ERK signaling pathway. Although the role of KAT7 in regulating cell proliferation and apoptosis in cancer cells has been previously reported 27,45, the precise mechanisms by which KAT7 regulates CRC tumorigenesis remain unclear. The MAPK pathway governs crucial cellular processes such as development, differentiation, proliferation, and cell death. Structured in a three-tier hierarchy (MAP3K, MAP2K, and MAPK), its signaling kinases are categorized into three families: JNK, P38, and ERK 39,46. Through RNA-seq and western blot validation, our data demonstrate that knockdown of KAT7 inhibits CRC tumorigenesis specifically through the MAPK/ERK pathway, rather than the P38 or JNK pathways. Additionally, the use of an ERK agonist rescues the decreased proliferation and activated apoptosis caused by KAT7 knockdown in CRC cells. Collectively, these results highlight the significance of histone acetyltransferases in promoting CRC progression through the MAPK/ERK signaling pathway.

Furthermore, our results suggest that KAT7 upregulates the MAPK/ERK signaling pathway through H3K14ac-binding MRAS transcriptional activation. Considering that MAPK/ERK is a widely recognized pathway with protumorigenic effects in colorectal tumorigenesis, our focus was on identifying the specific upstream components of the MAPK/ERK pathway influenced by KAT7. MRAS, a member of the RAS family, has recently been found to form a holophosphatase complex with SHOC2 and PP1C. This complex plays a crucial role in regulating RTK-RAS signaling by eliminating inhibitory phosphorylation on RAF family proteins, thereby enhancing MAPK signaling. Our study revealed that MRAS is highly expressed in CRC and shows a positive correlation with KAT7 expression levels. Moreover, CRC patients with high MRAS expression showed poorer prognosis. Overexpression of MRAS rescues the inhibition of the MAPK/ERK pathway, decreases cell proliferation, and activates apoptosis caused by KAT7 knockdown. KAT7 exhibits acetyltransferase activity and can acetylate both histone and nonhistone substrates in mammals. In our study, we found that KAT7 significantly promotes H3K14 acetylation in CRC cells. Furthermore, the acetyltransferase activity of KAT7 is crucial for MRAS gene transcription, MAPK/ERK pathway activation, and the survival of CRC cells. The ChIP-PCR experiment confirmed the binding between KAT7 and the promoter region of the MRAS. This binding enables KAT7 to promote H3K14 acetylation in the MRAS gene promoter region, thereby regulating MRAS transcription.

Finally, our *in vivo* experimental results demonstrate that knockdown of KAT7 in CRC cells significantly inhibits their growth in mice and suppresses their metastasis to the lungs. Additionally, KAT7 knockdown markedly extended the lifespan of the tumor-bearing mice. In summary, our findings identify KAT7 as a prospective target for CRC treatment. We have uncovered a mechanism by which KAT7 promotes CRC tumorigenesis through the H3K14ac/MRAS-MAPK/ERK pathway. The roles of KAT7 in CRC shed light on its broader regulation of human diseases.

## Conclusions

We found that KAT7 promotes the malignant phenotype of CRC through the H3K14ac/MRAS-MAPK/ERK pathway, making KAT7 a potential therapeutic target for CRC.

## Supplementary Material

Supplementary figures and tables.

## Abbreviations

HAT: Histone acetyltransferase
CRC: colorectal cancer
H3K14: H3 at lysine 14
HAT: histone acetyltransferase
HDAC: histone deacetylase
EdU: 5-ethynyl-20-deoxyuridine
B-NDG: NOD.CB17-Prkdcscid/l2rgtm1/Bcgen
ChIP: Chromatin immunoprecipitation
PARP: poly ADP-ribose polymerase
EMT: Epithelial-mesenchymal transition
GO: gene ontology
KEGG: Kyoto Encyclopedia of Genes and Genomes
