# Supplementary material for: Targeting KAT7 inhibits the progression of colorectal cancer
Source: Theranostics. 2025 Jan 2;15(4):1478–95. doi: 10.7150/thno.106085 (PMC11729548; doi:10.7150/thno.106085)
Supplement: Supplementary file 1 — Supplementary figures and tables. [file thnov15p1478s1.pdf]

Fig. S1

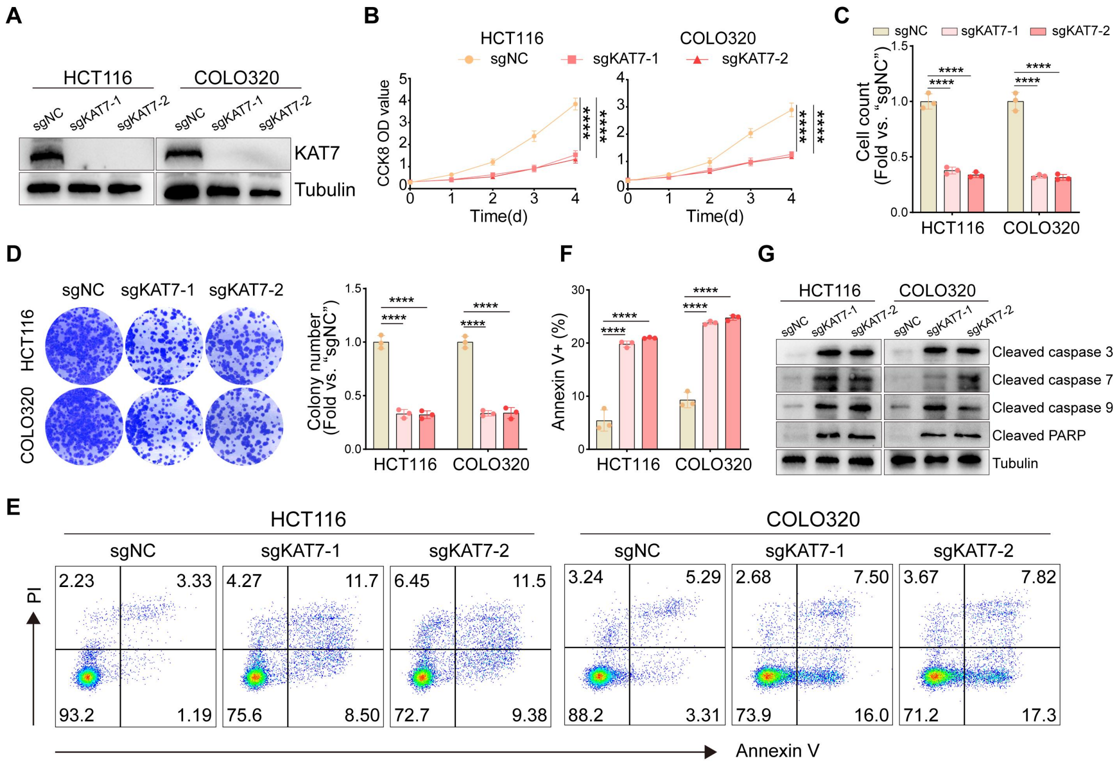

Figure S1. CRISPR/Cas9-induced KAT7 knockout suppresses CRC cell viability, proliferation, and promotes apoptosis

(A) Successful knockout of KAT7 in the indicated cells using the CRISPR/Cas9 system. **(B)** Knockout of KAT7 suppresses the viability of CRC cells. **(C)** CRC cells were treated as described in **(A)**, and cell counting was performed to observe cell proliferation. **(D)** After KAT7 knockout, the colony-forming ability of cells was reduced, as shown in colony photographs (left) and colony count statistics (right). **(E-F)** Flow cytometry analysis of apoptosis in the corresponding cells, represented by the flow cytometry scatter plot **(E)** and the statistical graph of apoptotic cell percentage **(F)**. **(G)** Increased expression of apoptosis-related proteins after KAT7 knockout.

Error bars represent the mean ± standard deviation (SD). Statistical significance was assessed using one-way ANOVA for experiments **(B-D, F)**. ****P < 0.0001.

Fig. S2

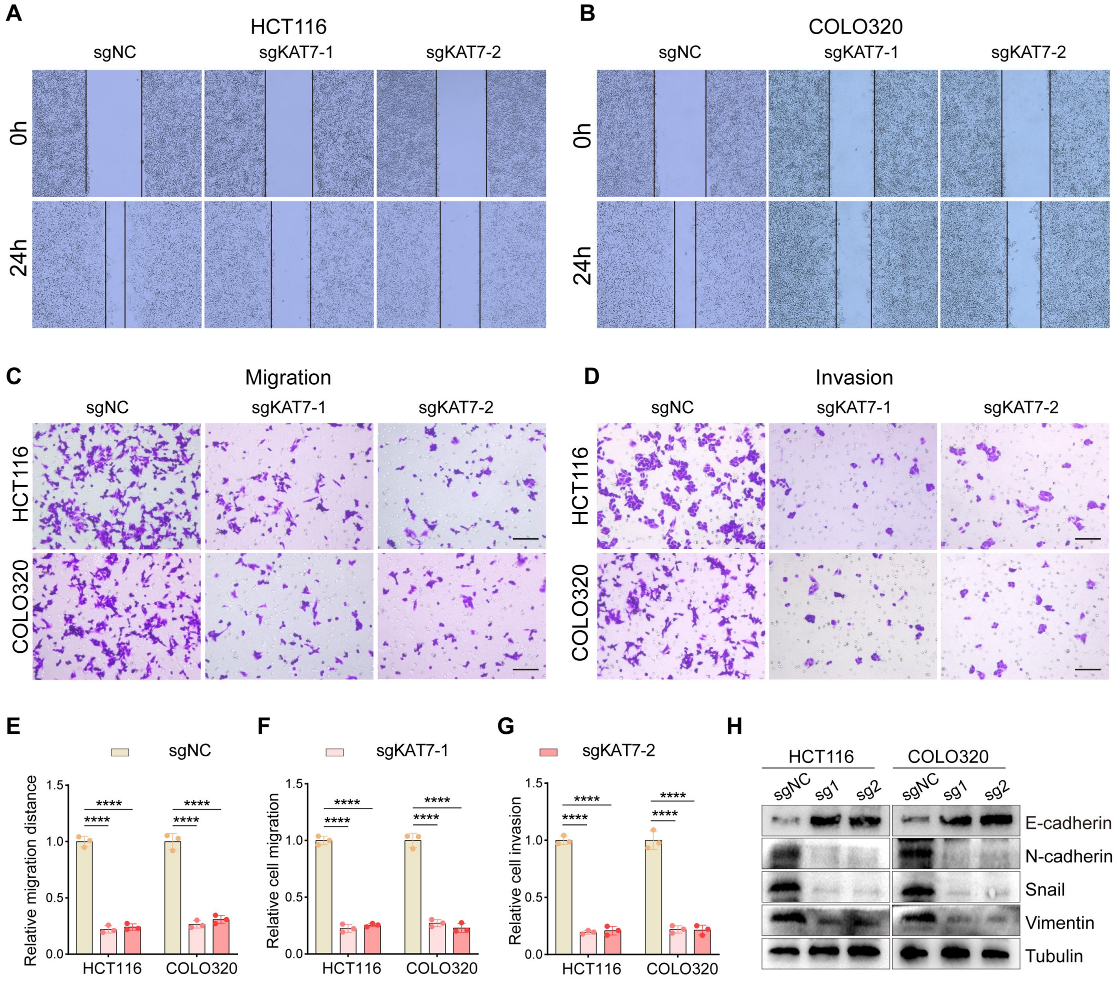

Figure S2. KAT7 knockout inhibits the migration and invasion of CRC cells. **(A-B)** Wound-healing assay assessing the impact of KAT7 knockout on the migration ability of CRC cells, including HCT116 cells **(A)** and COLO320 cells **(B)**. **(C)** Transwell chamber assay demonstrating significant inhibition of CRC cell migration after KAT7 knockout (scale bar = 100 μm). **(D)** KAT7 knockout suppresses CRC cell invasion (scale bar = 100 μm). **(E)** Statistical analysis of cell migration distance for the cells shown in **(A-B)**. **(F)** Statistical analysis of the number of migrated cells from

(C). (G) Statistical analysis of the relative quantity of invaded cells from (D). (H) Western blot analysis assessing the impact of KAT7 knockout on the expression levels of Epithelial-mesenchymal transition (EMT) -related proteins. Error bars represent the mean ± SD. Statistical significance was assessed using one-way ANOVA for experiments (E-G). ****P < 0.0001.

Fig. S3

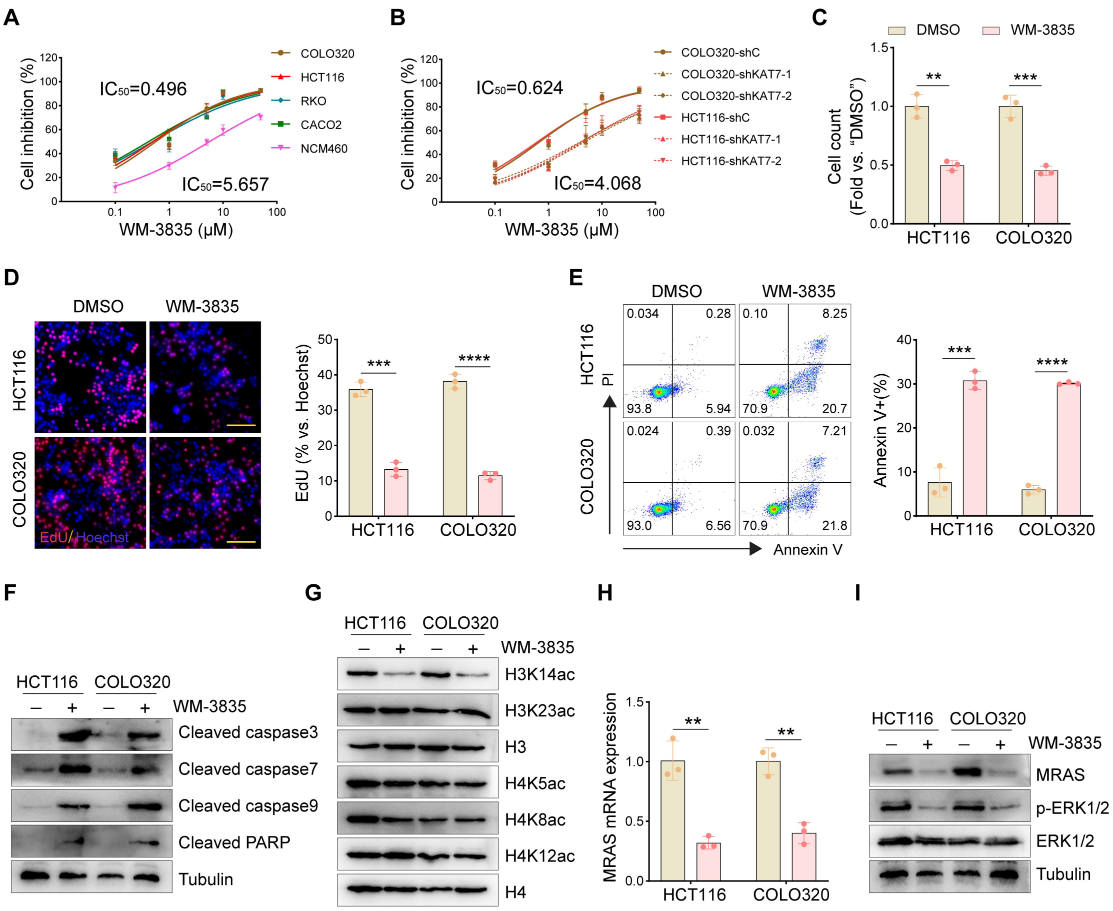

Figure S3. The anti-CRC effects of the KAT7 inhibitor WM-3835.

(A-B) Dose-response curves showing the inhibitory effects of WM-3835 on the indicated cells in vitro after 96 hours of treatment. **(C)** Cell counts of CRC cells treated with 0.1% DMSO or 0.5 μM WM-3835 and cultured for 96 hours. **(D)**

Proliferation of HCT116 and COLO320 cells treated as in (C) was assessed using EdU staining (Scale bar = 100 μm). **(E-F)** CRC cells treated as in (C) were analyzed for apoptosis using flow cytometry **(E)** and Western blotting **(F)**. **(G)** Western blot analysis showing the effects of WM-3835 on histone acetylation in CRC cells. **(H)** WM-3835 significantly inhibited the expression of the MRAS gene. **(I)** Western blot analysis of the effects of WM-3835 on the expression of the indicated proteins in CRC cells. Error bars represent the mean ± SD. Statistical significance was assessed using two-tailed unpaired student's *t* tests for experiments **(C-E, H)**. **P < 0.01, ****P < 0.001, ****P < 0.0001.

Table S1. The antibodies used in this study

Antibodies	Source	Identifier
KAT7	proteintech	13751-1-AP
GAPDH	proteintech	60004-1-Ig
Tubulin	proteintech	66031-1-Ig
cleaved caspase3	HUABIO	ET1602-47
cleaved caspase7	HUABIO	ER60002
cleaved caspase9	HUABIO	ER60008
cleaved PARP	HUABIO	ET1608-10
E-cadherin	proteintech	20874-1-AP
N-cadherin	proteintech	22018-1-AP
Snail	proteintech	13099-1-AP
Vimentin	proteintech	10366-1-AP
p-ERK1/2	HUABIO	ET1610-13
ERK1/2	HUABIO	ET1601-29
p-JNK	HUABIO	ET1609-42
JNK	HUABIO	ET1601-28
p-P38	HUABIO	ER2001-52
P38	HUABIO	ET1702-65
MRAS	proteintech	14213-1-AP
H3K14ac	Cell Signaling Technology	7627
H3K23ac	Bioworld	BS70005
H3	HUABIO	M1309-1
H4K5ac	bioss	bs-10721R
H4K8ac	Bioworld	BS74016
H4K12ac	ABcam	ab177793
H4	proteintech	16047-1-AP
Ki67	Servicebio	GB121141-100

Table S2. Summary of the primer sequences used in this study

	**qRT-PCR**	
gene	Forward 5'-3'	Reverse 5'-3'
β-actin	TGGCACCCAGCACAATGAA	CTAAGTCATAGTCCGCCTAGAAGCA
MRAS	CAACAAGGTCGATTTGATGCACT	TTTCTTTTCCTTGCTCCCTGGTGA
FGF9	CTAAACGGCACCAGAAATTCACA	TTGGCTTAGAATATCCTTATACAGT
EPHA2	TTCAGCCACCACAACATCATCCG	GCTGAACTCGCCATCCTTCTCCC
PDGFB	GCAAGCACCGGAAATTCAAGC	TCTCCTTCAGTGCCGTCT
MECOM	TTCAAAGACAAAGTAAGCCCTC	TCTGCTCCTCTAAAGATGGTGA
PRKACB	TTAAAGGCAGAACTTGGACA	CTGCCATTTCATAGATTAGCAC
IGF1R	CCGCAACCTGACCATCAAAGCA	CTTTAGTCCCCGTCACTTCCTCC
IL1RAP	ATGAAACTCCCAGTGCAT	GCCCATATACCAAGTGATAGTCG
TGFA	ATTTTAATGACTGCCCAGATTCCC	CCAACGTACCCAGAATGGCAGA
ANGPT2	AACACAAATAAGTTCAACGGCAT	AATAGCCTGAGCCTTTCCAGT
	**ChIP-PCR**	
gene	Forward 5'-3'	Reverse 5'-3'
MRAS	GATGTACAGATCCCTGCCCG	AGGTTGGGTGCCATCAAGAG
